# Validating a Framework for Coding Patient-Reported Health Information to the Medical Dictionary for Regulatory Activities Terminology: An Evaluative Study

**DOI:** 10.2196/medinform.9878

**Published:** 2018-08-21

**Authors:** Sonja Brajovic, David A Blaser, Meaghan Zisk, Christine Caligtan, Sally Okun, Marni Hall, Carol A Pamer

**Affiliations:** ^1^ Office of Surveillance and Epidemiology Center for Drug Evaluation and Research US Food And Drug Administration Silver Spring, MD United States; ^2^ PatientsLikeMe Cambridge, MA United States; ^3^ Center for Advanced Evidence Generation Real World Insights IQVIA Rockville, MD United States

**Keywords:** adverse drug events, Food and Drug Administration, MedDRA, patient-generated health data, PatientsLikeMe, vocabulary, controlled, data curation

## Abstract

**Background:**

The availability of and interest in patient-generated health data (PGHD) have grown steadily. Patients describe medical experiences differently compared with how clinicians or researchers would describe their observations of those same experiences. Patients may find nonserious, known adverse drug events (ADEs) to be an ongoing concern, which impacts the tolerability and adherence. Clinicians must be vigilant for medically serious, potentially fatal ADEs. Having both perspectives provides patients and clinicians with a complete picture of what to expect from drug therapies. Multiple initiatives seek to incorporate patients’ perspectives into drug development, including PGHD exploration for pharmacovigilance. The Food and Drug Administration (FDA) Adverse Event Reporting System contains case reports of postmarketing ADEs. To facilitate the analysis of these case reports, case details are coded using the Medical Dictionary for Regulatory Activities (MedDRA). PatientsLikeMe is a Web-based network where patients report, track, share, and discuss their health information. PatientsLikeMe captures PGHD through free-text and structured data fields. PatientsLikeMe structured data are coded to multiple medical terminologies, including MedDRA. The standardization of PatientsLikeMe PGHD enables electronic accessibility and enhances patient engagement.

**Objective:**

The aim of this study is to retrospectively review PGHD for symptoms and ADEs entered by patients on PatientsLikeMe and coded by PatientsLikeMe to MedDRA terminology for concordance with regulatory-focused coding practices.

**Methods:**

An FDA MedDRA coding expert retrospectively reviewed a data file containing verbatim patient-reported symptoms and ADEs and PatientsLikeMe-assigned MedDRA terms to determine the medical accuracy and appropriateness of the selected MedDRA terms, applying the International Council for Harmonisation MedDRA Term Selection: Points to Consider (MTS:PTC) guides.

**Results:**

The FDA MedDRA coding expert reviewed 3234 PatientsLikeMe-assigned MedDRA codes and patient-reported verbatim text. The FDA and PatientsLikeMe were concordant at 97.09% (3140/3234) of the PatientsLikeMe-assigned MedDRA codes. The 2.91% (94/3234) discordant subset was analyzed to identify reasons for differences. Coding differences were attributed to several reasons but mostly driven by PatientsLikeMe’s approach of assigning a more general MedDRA term to enable patient-to-patient engagement, while the FDA assigned a more specific medically relevant term.

**Conclusions:**

PatientsLikeMe MedDRA coding of PGHD was generally comparable to how the FDA would code similar data, applying the MTS:PTC principles. Discordant coding resulted from several reasons but mostly reflected a difference in purpose. The MTS:PTC coding principles aim to capture the most specific reported information about an ADE, whereas PatientsLikeMe may code patient-reported symptoms and ADEs to more general MedDRA terms to support patient engagement among a larger group of patients. This study demonstrates that most verbatim reports of symptoms and ADEs collected by a PGHD source, such as the PatientsLikeMe platform, could be reliably coded to MedDRA terminology by applying the MTS:PTC guide. Regarding all secondary use of novel data, understanding coding and standardization principles applied to these data types are important.

## Introduction

Patients describe their medical experiences differently compared with how clinicians or researchers would describe their observations. The interpretation of data gathered from patients is typically based on a clinician’s perspective of how a patient is feeling or functioning or what is of most concern to a clinician. However, a clinician’s impression may differ from a patient’s experience and therefore may not be an accurate or complete interpretation [1-3]. For example, a patient may find a nonserious, known adverse drug event (ADE) to be an ongoing, daily concern, which impacts tolerability and adherence. Clinicians, however, must be vigilant for medically serious or potentially fatal ADEs. Having both perspectives can provide patients and clinicians with a complete picture of what to expect from drug therapy options for a given medical condition.

With new advances in technology, the availability of and interest in patient-generated health data (PGHD) have grown steadily. PGHD are distinct from data generated in clinical settings, such as within clinical trials or during encounters with health care practitioners. Most importantly, patients directly report and record these data and are responsible for sharing and distributing them [4]. The internet democratized access to and sharing of health information, leading to the creation of Web-based patient networks. The growing popularity and advancement of mobile sensors, wearable devices, and smartphones have created enormous opportunity for innovative approaches to understanding, managing, and improving health through the collection and application of PGHD [5]. Several ongoing initiatives incorporate patients’ perspectives into drug development processes. For example, the Patient-Centered Outcomes Research Institute was established to address challenges with traditional research and integrate patients as key stakeholders and partners in the research process [6]. In addition, the US Food and Drug Administration (FDA) Prescription Drug User Fee Act V mandated public engagement through patient-focused drug development workshops and development of patient-reported outcomes to better understand the impact of symptoms and treatments on patients’ lives [7]. The 21st Century Cures Act, among other provisions, also requires the FDA to consider real-world evidence and patient-experience data in its review of drugs and devices [8].

The FDA Adverse Event Reporting System (FAERS) contains case reports of postmarketing adverse events submitted to the FDA Center for Drug Evaluation and Research and Center for Biologics Evaluation and Research [9]. The FDA uses these reports to generate and evaluate signals of potential adverse reactions to drugs and biologics [10]. To facilitate searching and analysis of the case reports in FAERS, all suspected ADEs, such as medical diagnoses and stand-alone signs or symptoms, as well as medication errors and product quality issues included in case narratives, are coded using the Medical Dictionary for Regulatory Activities (MedDRA). MedDRA is a terminology endorsed by the International Council on Harmonisation (ICH) to be used by regulators and the pharmaceutical industry to codify ADEs reported with the use of medical products [11]. The ICH MedDRA Term Selection: Points to Consider (MTS:PTC) document provides valuable guidance on best practices for term selection and promotes accuracy and consistency in coding [12]. Pharmaceutical companies precode the reported suspected adverse reactions to drugs or biologics to MedDRA according to the MTS:PTC guide. The FDA then samples and reviews the submitted MedDRA codes for coding quality, medical accuracy, and alignment with MTS:PTC. Moreover, the FDA applies the MTS:PTC guide to internally code FAERS case reports received directly through MedWatch, the FDA’s voluntary reporting system for health care professionals, consumers, and patients [13]. Medically accurate and thorough MedDRA coding is an essential prerequisite for subsequent reliable and comprehensive retrieval of pertinent cases through electronic querying by MedDRA codes. While MedDRA terminology is critical for searching databases and conducting data mining activities, these terms do not necessarily fully reflect a patient’s experience.

PatientsLikeMe (PLM) is a Web-based network where patients report, track, share, and discuss their health information. PLM captures PGHD through free-text entries and structured data fields; these data have previously been used to conduct research from patients’ perspectives [14-16]. The structured fields for entering data, such as patients’ conditions, symptoms, or treatments, are coded by PLM to several established medical terminology systems, including MedDRA, to increase its usefulness for research and enable interoperability with external systems.

This study aimed to evaluate the concordance and discordance of MedDRA coding results of verbatim patient reports in a structured PGHD setting compared with how the same submissions would be coded based on the MTS:PTC coding guide, the current standard for regulatory settings.

## Methods

We conducted a retrospective evaluation of PLM MedDRA coding of PGHD of signs, symptoms, and ADEs collected in structured data fields on the PLM platform and compared this with how the same PGHD data would be coded to MedDRA applying the MTS:PTC. Medical terminology systems, such as MedDRA, are typically developed to be used by health care professionals, regulators, pharmaceutical industry, and researchers. Although MedDRA contains “lay-person” terms, it does not contain all the language familiar to most patients. To help bridge the gap between clinical terminology in MedDRA and patient-friendly language, the PLM terminology was created using the words and phrases reported by patients. The PLM terminology, which is maintained and curated by a team of medical professionals, consists of several types of medical entities, each of which is coded to one or more external medical terminology systems (Table 1).

**Figure 1:**
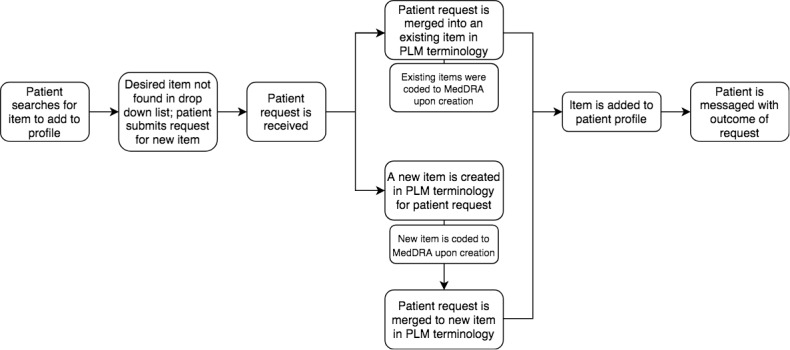
Diagram of information flow for patient submissions. MedDRA: Medical Dictionary for Regulatory Activities; PLM: PatientsLikeMe.

Patients who wish to share and track their personal health history can search the PLM terminology to find an item (eg, condition, symptom, and treatment) to add to their profile. As patients enter text in the search field, a range of possible matches in the PLM terminology are suggested; patients may choose an appropriate match or submit a request to add a new item. A medical professional at PLM reviews the request and determines whether it can be merged to an existing item in the PLM terminology (Figure 1).

When a new item is added to the PLM terminology, a detailed entry is created to describe and distinguish the concept appropriately (Figure 1). For each new item, PLM writes an appropriate patient-facing description, codes the item to appropriate medical terminologies, and sets any relevant customizations to guide data entry for future patients. Once this process is complete, the curated PGHD becomes a new item in the PLM terminology and appears on the patient profile. Finally, PLM sends a message to notify the patient that the new item has been added to the PLM terminology. When a verbatim patient report is curated for any reason, the patient receives a message alerting them to the change and providing them with the option to accept or reject the change and to request the original entry be retained.

A data file was generated containing PGHD for symptoms and ADEs entered through the PLM platform from January 1, 2013 to September 1, 2015; this file contained the patient’s verbatim text, the item(s) it was merged to in the PLM terminology, and the MedDRA term associated with the item(s) as assigned by PLM. Only PGHD from patients with an active account from the United States were included in the data file. Notably, the data file did not include any patient-level, personally identifiable information. A MedDRA terminology expert reviewer from the FDA evaluated each record in the file for accuracy and specificity of the MedDRA terminology coding, applying the MTS:PTC guide. MedDRA has a hierarchical structure with 5 levels as follows: System Organ Class (SOC), High-Level Group Term (HGLT), High-Level Term (HLT), Preferred Term (PT), and Lowest-Level Term (LLT). The PT level is considered a distinct descriptor (single medical concept) [10]. The LLTs under a PT are synonyms and lexical variants of the PT; each LLT is linked to only one PT. LLTs reflect how information might be reported and represent the coding level. Symptoms and ADEs in the PLM database are coded to an LLT, as per the established coding practice.

In this study, the FDA reviewer evaluated the appropriateness of the MedDRA term(s) associated with the patients’ verbatim text. The FDA reviewer provided written comments for each record indicating one of the following coding results categories:

- MedDRA coding appropriate: Both the FDA and PLM reviewer agreed that the selected MedDRA term was appropriate for the PGHD verbatim.
- Incorrect MedDRA coding: The MedDRA term assigned by PLM was not appropriate.
- Missed concept: A component of the PGHD verbatim entry was not coded to a MedDRA term.
- Outdated MedDRA version: Reviewer recommended a more specific MedDRA term, but it was not available in the MedDRA version used by PLM.
- Duplicate: A unique record was listed more than once in the extracted dataset. These records were not included in the final analysis.
- Unable to assess: PLM’s coding decision for these records was based on additional information available as free text on the patient’s profile and private message communication between PLM and the patient. Specifically, PLM engages with patients through private messages to learn more about patients’ experiences to capture the information as reported accurately; this additional information was not available in the data file provided to the FDA for review. Thus, these records could not be properly evaluated for coding accuracy and were excluded from the final analysis.

**Table 1:** Coding for data elements of the PatientsLikeMe terminology.

Medical entity type	Examples	Terminology
Condition	Multiple sclerosis, epilepsy, traumatic brain injury, major depressive disorder	Systematized Nomenclature of Medicine-Clinical Terms (SNOMED CT), International Classification of Diseases-10 (ICD10), Medical Dictionary for Regulatory Activities (MedDRA)
Symptom	Depressed mood, anxious mood, fatigue, insomnia, pain	SNOMED CT, ICD10, MedDRA, International Classification of Functioning, Disability and Health
Treatment	Gabapentin, vitamin D, physical therapy, cognitive behavioral therapy	RxTerms
Side effect	May be coded to a condition or symptom	See above for Terminology
Treatment purpose	May be coded to a condition or symptom	See above for Terminology
Hospitalization reason	May be coded to a condition, symptom, or treatment	See above for Terminology

For each included record, other than those classified as “MedDRA coding appropriate,” the reviewer provided a rationale for disagreement and proposed an alternative MedDRA code. Together, the FDA and PLM reviewers discussed each discordant record, reached consensus on the category, and documented the reasons for discordant coding.

## Results

The data file from PLM contained 3349 submissions. A total of 115 items were identified as “duplicates” (n=20) or “unable to assess” (n=95) and were excluded from the final analysis. Examples of entries in this category are: “loss of blood,” LLT *Rectal bleeding* (patient clarified the source of blood loss); “systemic nerve overstimulation,” LLT *Essential tremor* (patient clarified); “blood pressure,” LLT *Blood pressure high* (patient reported high blood pressure just as “blood pressure”); “hose hurts my ears,” LLT *Skin irritation* [a patient with chronic obstructive pulmonary disorder on supplemental oxygen therapy].

A total of 3234 MedDRA-coded items remained for the final analysis. The expert reviewer determined that these verbatim terms could be MedDRA-coded as per the patient-reported submission and found that MedDRA coding was appropriate in 97.09% (3140/3234) of cases (Table 2). These reported verbatim terms ranged from specific medical terms (“intercostal neuralgia” and “coccydynia”), to less specific terms (“balance problems” and “blood clots in legs”) and personal communications (“just didn’t feel right” and “zombie mommy”). Table 3 illustrates examples of differences between the PLM and FDA approach to coding based on MTS:PTC.

In Table 3, example 1 wherein the patient submission of “Pruritis” was merged into the existing PLM term “Itching,” demonstrates an instance in which more patient-friendly language is preferred by PLM. In the PLM terminology, the term “itching” encompasses all entries related to “pruritus,” “itch,” “itchy skin,” and “itchiness.” After discussion, the FDA reviewer agreed that the coding was appropriate at the PT level, as both LLT *Pruritus* and LLT *Itching* roll up to the same PT *Pruritus*. This example illustrates that the language used by patients can range from informal to highly technical.

Example 2 is an instance of PLM coding an item to more specific MedDRA terms than the FDA. The PLM terminology contains separate terms for a symptom affecting different body area; this approach enables patients to track and monitor a symptom on each body area individually. Thus, PLM chose to split the patient submission “rash on chest & neck” into the individual symptoms “rash on chest” and “rash on neck.” “Rash on chest” was mapped to the LLT *Skin rash*, whereas “rash on neck” was coded to the LLT *Neck rash*. However, the FDA coding practice is to code “rash on chest and neck” to a single MedDRA term, LLT *Rash,* and consider it a single event as the term for both “rash on chest” and “rash on neck” roll up to the same PT in the MedDRA hierarchy.

**Table 2:** Results of the Food and Drug Administration Medical Dictionary for Regulatory Activities (MedDRA) coding review (N=3234^a^).

Category	Records, n (%)
Coding appropriate	3140 (97.09)
Incorrect coding	45 (1.39)
Missed concept	38 (1.18)
Outdated MedDRA version	11 (0.34)

^a^Excludes “duplicates” (n=20) and “unable to assess” (n=95).

**Table 3:** Examples of Medical Dictionary for Regulatory Activities (MedDRA) coding from the analysis.

#	Patient submission	PLM^a^ action	PLM MedDRA coding		FDA^b^ reviewer assessment		Outcome	
			LLT^c^	PT^d^				
1	Pruritis	Merged into PLM symptom “Itching”	Itching	Pruritus		Coding appropriate		N/A^e^
2	Rash on chest & neck	Merged into PLM symptoms “Rash on chest” and “Rash on neck”	Skin rash; neck rash	Rash		Coding appropriate		N/A
3	Drop foot from surgery	Merged into PLM symptom “Foot drop”	Foot drop	Peroneal nerve palsy		Coding appropriate; however, more specific term is available. Recommend: LLT Peroneal nerve palsy postoperative; PT Peroneal nerve palsy postoperative		Coding was not changed because of the PLM conceptual approach to coding to more general term
4	Sexual dysfunction/no libido	Merged into PLM symptom “Sexual dysfunction”	Sexual dysfunction	Sexual dysfunction		Coding appropriate		N/A
5	Irrational emotions	Merged into PLM symptom “Irrational emotions”	Mixed disturbance of conduct and emotions	Antisocial behavior		Incorrect coding; recommend coding to: LLT Emotional disorder; PT Emotional disorder		Coding updated
6	Cardiac arrhythmia	Merged into PLM symptom “Irregular heartbeat (cardiac arrhythmia)”	Heartbeats irregular	Heart rate irregular		Incorrect coding; recommend coding to: LLT Cardiac Arrhythmia; PT Arrhythmia		Coding updated
7	Increased INR^f^	Merged into PLM symptom “High INR”	INR abnormal	INR abnormal		Incorrect coding; recommend coding to: LLT INR increased; PT INR increased		Coding updated
8	I was stumbling, falling and tripping, my thinking process was very slow and my memory was failing too	Merged into PLM symptoms “Memory problems,” “Slowed thinking,” and “Tripping and stumbling”	Memory disturbance; slowed thinking; muscular incoordination	Memory impairment; Bradyphrenia; coordination abnormal		Missed concept. Report of “falling” is missing; falls are an important patient safety issue which should be captured. For stumbling and tripping, there is LLT Stumbling and LLT Gait tripping, both under PT Gait disturbance		Coding updated to include additional information
9	Back pain and nausea and vomiting	Merged into PLM symptoms “Nausea and vomiting” and “Back pain”	Vomiting; back pain	Vomiting; back pain		Missed concept (nausea)		PLM symptom “Nausea and vomiting” retired. The separate symptoms “Nausea” and “Vomiting” remain available
10	Gluten sensitivity	Merged into PLM symptom “Gluten intolerance”	Gluten intolerance	Celiac disease		Outdated MedDRA version; coding appropriate for this version of MedDRA, but more appropriate terms available in newer versions. Recommend coding to: LLT Gluten sensitivity; PT Gluten sensitivity		Coding updated after upgrade to more recent MedDRA version
11	Shocked by electricity house	Merged into PLM symptom “Seizures (grand mal or tonic–clonic)”	Tonic-clonic seizures	Grand mal convulsion		Unable to assess. Not clear from information how this could be determined		Communications with patient indicated this was reference to seizures

^a^PLM: PatientsLikeMe.

^b^FDA: Food and Drug Administration.

^c^LLT: lowest-level term.

^d^PT: preferred term.

^e^N/A: not applicable.

^f^INR: international normalized ratio

Example 3 shows that in some other instances, PLM symptoms are intentionally coded into more general MedDRA terms to help connect patients with similar experiences. PLM members reporting “drop foot” are assigned the same symptom in the PLM terminology regardless of the cause. Thus, the patient submission “drop foot from surgery” was merged into “Foot drop,” an existing symptom in PLM terminology. “Foot drop” was then coded to the MedDRA LLT *Foot drop*, which is subsumed under the PT *Peroneal nerve palsy.* The FDA reviewer noted that although this was an acceptable choice for the concept, the more specific LLT-PT *Peroneal nerve palsy postoperative* is preferable because it captures the additional information that the foot drop was consequent to a surgery. However, all patients reporting “drop foot” are assigned the same symptom in the PLM terminology regardless of the cause.

Example 4 demonstrates how PLM aggregates experiences related to “sexual dysfunction,” even though this likely refers to distinct manifestations for different genders. Patients have the option to add more specific symptoms, which are coded to more specific MedDRA terms when appropriate, such as “impotence” (coded to LLT *Impotence*) or “loss of sex drive” (coded to LLT *Libido loss*).

Examples 5, 6, and 7 illustrate the items in which the PLM-assigned MedDRA terms did not align with the MTS:PTC guide. In example 5, the selected MedDRA term was not medically accurate for the patient submission of “irrational emotions.” Although the LLT *Mixed disturbance of conduct and emotions* appears at first to be a reasonable option, it is subsumed under the PT *Antisocial behavior*, which the FDA reviewer determined was not an appropriate code. PLM agreed and updated the coding for this symptom as per the reviewer’s recommendation and investigated what may have contributed to the selection of the incorrect term. The likely reason is that only the name of the MedDRA LLT was visible during the data entry process for adding or editing a symptom in PLM terminology. The LLT’s association to a PT and higher levels in the MedDRA’s hierarchy was not displayed. When adding the new symptom “irrational emotions” to the PLM terminology, the original PLM coder would not have likely selected the LLT *Mixed disturbance of conduct and emotions* if they had been aware of its associated MedDRA PT. Example 6 shows how 2 related medical concepts, irregular heartbeats and cardiac arrhythmias, were considered as a single item in the PLM terminology. PLM codes certain symptoms with both the clinical terminology and more patient-friendly terminology to facilitate patients’ tracking their condition and connecting with other PLM patients with similar conditions. However, the MTS:PTC guide states that if both a diagnosis (eg, cardiac arrhythmia) and its characteristic signs or symptoms (eg, irregular heartbeats) are reported, the MedDRA term for the more definitive diagnosis should be selected. PLM has since updated its coding process to reflect this rule. Example 7 was coded incorrectly because it only captured the laboratory result as abnormal, rather than a directional change as specified in the patient submission. In this case, PLM also updated the coding to reflect the increased laboratory value.

Examples 8 and 9 are both instances in which a concept was missed by PLM’s coding, although the reasons for missing the concepts are distinct. In example 8, an additional MedDRA term should have been selected. In example 9, the missing concept, “nausea,” is captured in the name of the PLM symptom “nausea and vomiting,” but the assigned term (vomiting) does not capture this additional information. As a result, the symptom named “nausea and vomiting” was retired from patient search in the PLM terminology; patients can now add “nausea” and “vomiting” as separate and distinct symptoms to their profile.

In example 10, the FDA reviewer identified a more appropriate term. Further investigation of this example and other similar instances revealed that the recommended terms were available only in more recent MedDRA versions. Once PLM integrated the most recent MedDRA version into the platform, this coding was updated.

Example 11 demonstrates 1 of the 95 items that were flagged as “unable to assess” and had been excluded from the final evaluation. In these instances, the record available to the FDA reviewer did not contain all the information PLM had when assigning the MedDRA term, which was necessary to determine if the coding was appropriate.

## Discussion

### Principal Findings

This examination of coding of PLM PGHD to the MedDRA terminology revealed high concordance (3140/3234, 97.09%) between how PLM PGHD were coded to MedDRA and how the same submissions would be coded to MedDRA based on the MTS:PTC guide. The remaining 2.91% (94/3234) discordant coding instances revealed some important conceptual differences. Namely, the coding for regulatory purposes focuses on capturing the most specific information reported to a postmarketing safety surveillance program for ADEs and medication errors. Coding in the PLM scenario, however, requires first coding PGHD to patient-friendly terms in the curated PLM terminology and then coding the PLM terminology to established medical terminologies such as MedDRA to organize and aggregate medical information. In some instances, PLM codes to a more generalized MedDRA term to facilitate patients with similar symptoms or conditions in more easily finding each other on the PLM platform and sharing their experiences. For example, a patient may report a specific term such as “petit mal seizure,” on the PLM platform, but the reported concept would be grouped under a generalized term in the PLM terminology (“seizure,” LLT *Seizure*), whereas the same verbatim event (“petit mal seizure”) would be coded with specificity in FAERS (LLT *Petit mal*). PGHD data, if they are to serve the dual purposes of facilitating patient discussion and allowing for adverse event detection, need to have sufficient flexibility in coding to achieve the former goal while retaining the general adherence to the medical concepts that underlie coding in the first place. Users of these data need to understand the coding approach to optimize their data retrieval strategy.

This analysis reinforced the importance of several best practices. For example, when selecting a MedDRA term, it is essential to view the LLT-PT association and preferably the entire MedDRA hierarchy for the selected LLT to determine whether the term is, indeed, the most appropriate one. In addition, optimal coding results are achieved when using the latest available MedDRA version. It is important to conduct data coding quality reviews at regularly scheduled intervals.

### Limitations

Only a portion of the PLM MedDRA coding terminology was assessed to keep the dataset to a reasonable length. In addition, the PLM platform contains more information than the FDA reviewer had available in the dataset. Free-text information, which may have been private message communications about PGHD or information on the patient profile, was not shared with the FDA reviewer as it could have included personally identifiable information. Information from other structured sections of the profile (such as previously reported ADEs, symptoms, and treatments) was also available to PLM during the process of curating the PGHD but was not included in the data file provided to the FDA reviewer.

### Conclusions

This review demonstrates that PGHD consisting of signs, symptoms, and ADE data entered by patients in curated structured fields can be reliably coded to the MedDRA terminology and that the coding of these data by PLM is generally aligned with MTS:PTC principles. Understanding the coding purpose and approach is informative for the optimization of data retrieval strategy. These findings suggest that efficient electronic searching and aggregation of PGHD might be possible when consistent, systematic curation processes are applied to PGHD as they are reported by patients. This standardization makes PGHD more electronically accessible and therefore elevates the visibility and importance of events patients find most significant.

## Abbreviations

ADE: adverse drug event
FAERS: FDA Adverse Event Reporting System
FDA: Food and Drug Administration
HGLT: high-level group term
HLT: high-level term
ICH: International Council on Harmonisation
INR: international normalized ratio
LLT: lowest-level term
MedDRA: Medical Dictionary for Regulatory Activities
MTS:PTC: MedDRA Term Selection: Points to Consider
PGHD: patient-generated health data
PLM: PatientsLikeMe
PT: preferred term
SOC: system organ class

## References

[1] Basch E (2010). The missing voice of patients in drug-safety reporting. N Engl J Med.

[2] Blenkinsopp A, Wilkie P, Wang M, Routledge PA (2007). Patient reporting of suspected adverse drug reactions: a review of published literature and international experience. Br J Clin Pharmacol.

[3] Hakobyan L, Haaijer-Ruskamp FM, de ZD, Dobre D, Denig P (2011). A review of methods used in assessing non-serious adverse drug events in observational studies among type 2 diabetes mellitus patients. Health Qual Life Outcomes.

[4] Shapiro M, Johnston D, Wald J, Mon D (2012). Patient-generated health data: white paper prepared for the Office of the National Coordinator for Health IT.

[5] Piwek L, Ellis DA, Andrews S, Joinson A (2016). The Rise of Consumer Health Wearables: Promises and Barriers. PLoS Med.

[6] Clancy C, Collins FS (2010). Patient-Centered Outcomes Research Institute: the intersection of science and health care. Sci Transl Med.

[7] US 112th Congress (2012). Public Law 112–144.

[8] Burwell SM (2015). Setting value-based payment goals - HHS efforts to improve U.S. health care. N Engl J Med.

[9] US Food Drug Administration.

[10] Woodcock J, Behrman RE, Dal PGJ (2011). Role of postmarketing surveillance in contemporary medicine. Annu Rev Med.

[11] MedDRA: the Medical Dictionary for Regulatory Activities MedDRA Hierarchy.

[12] The International Council for Harmonisation of Technical Requirements for Pharmaceuticals for Human Use ICH MedDRA Term Selection: Points to Consider.

[13] Kessler DA (1993). Introducing MEDWatch. A new approach to reporting medication and device adverse effects and product problems. JAMA.

[14] de LLC, Dimova S, Mueller K, Phillips G, Durgin TL, Wicks P, Borghs S (2016). PatientsLikeMe® Online Epilepsy Community: Patient characteristics and predictors of poor health-related quality of life. Epilepsy Behav.

[15] Little M, Wicks P, Vaughan T, Pentland A (2013). Quantifying short-term dynamics of Parkinson's disease using self-reported symptom data from an Internet social network. J Med Internet Res.

[16] DasMahapatra P, Raja P, Gilbert J, Wicks P (2017). Clinical trials from the patient perspective: survey in an online patient community. BMC Health Serv Res.

